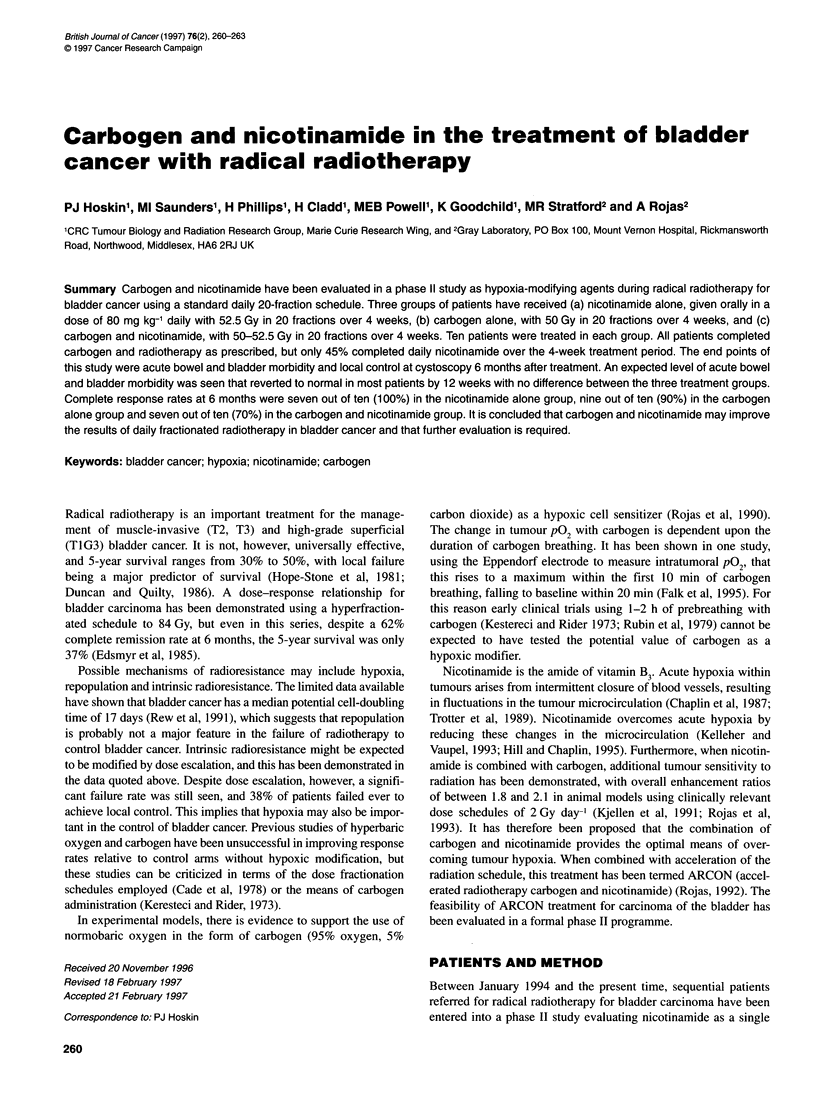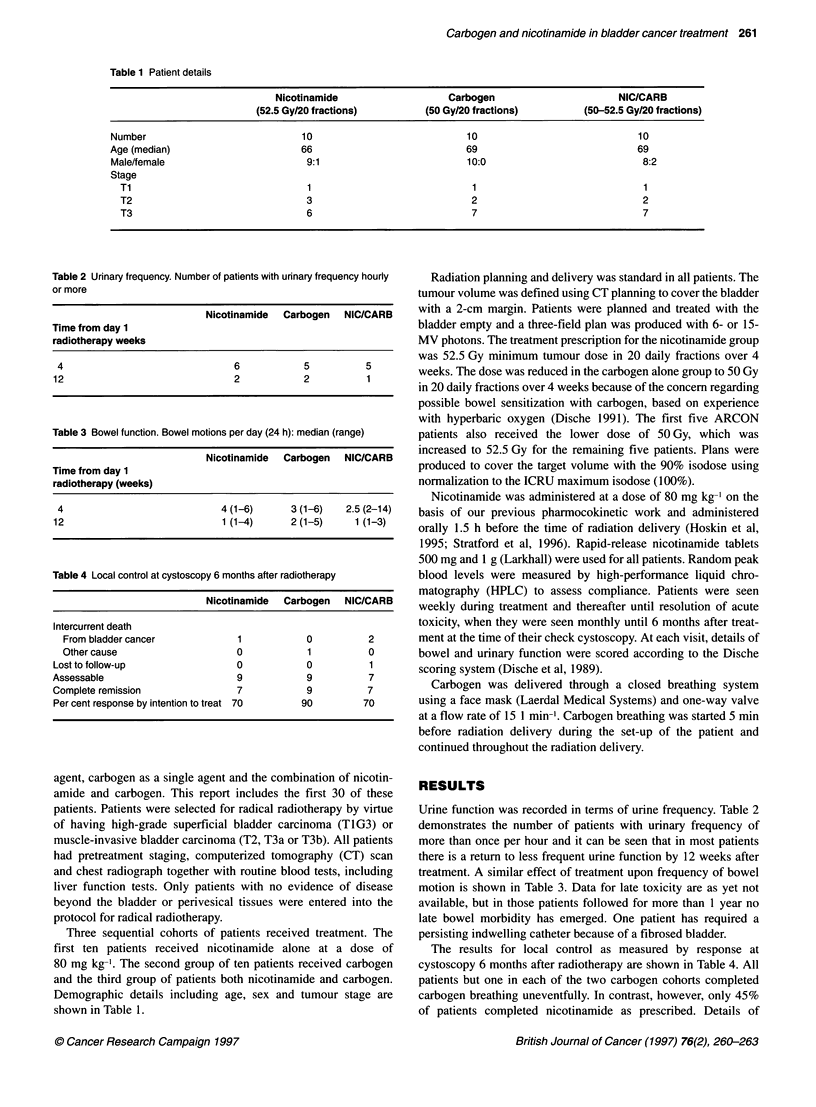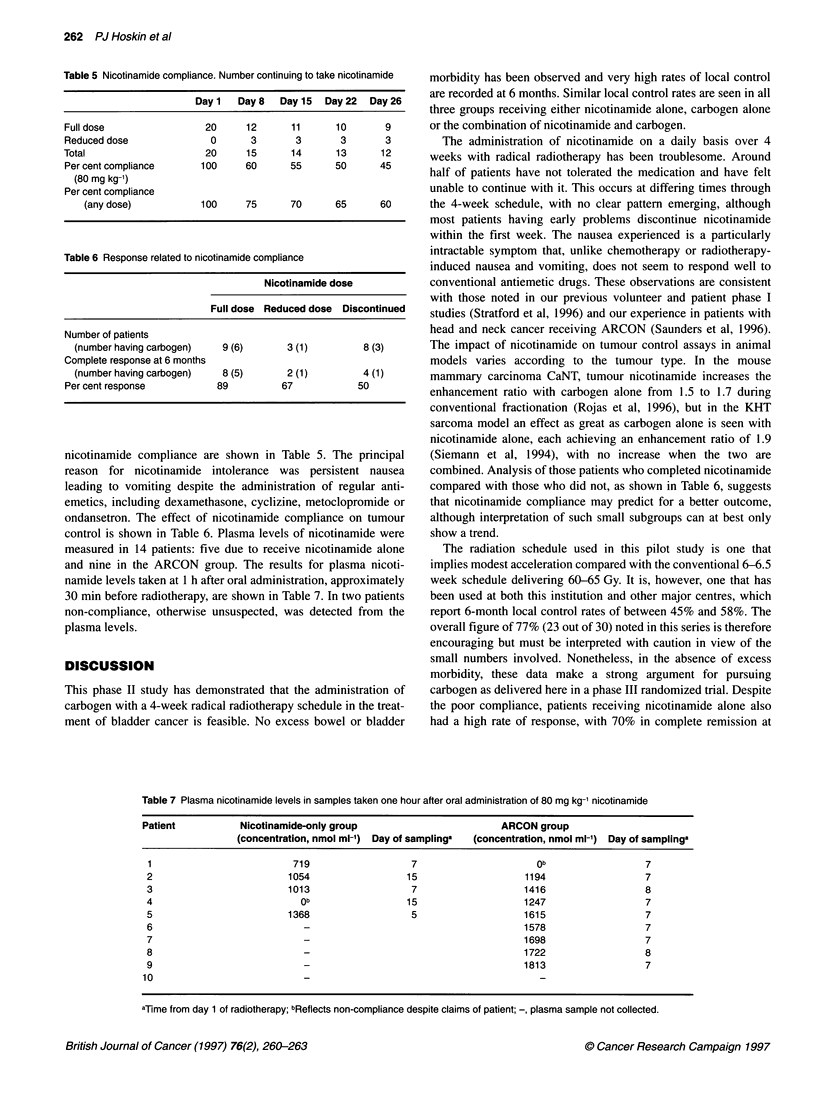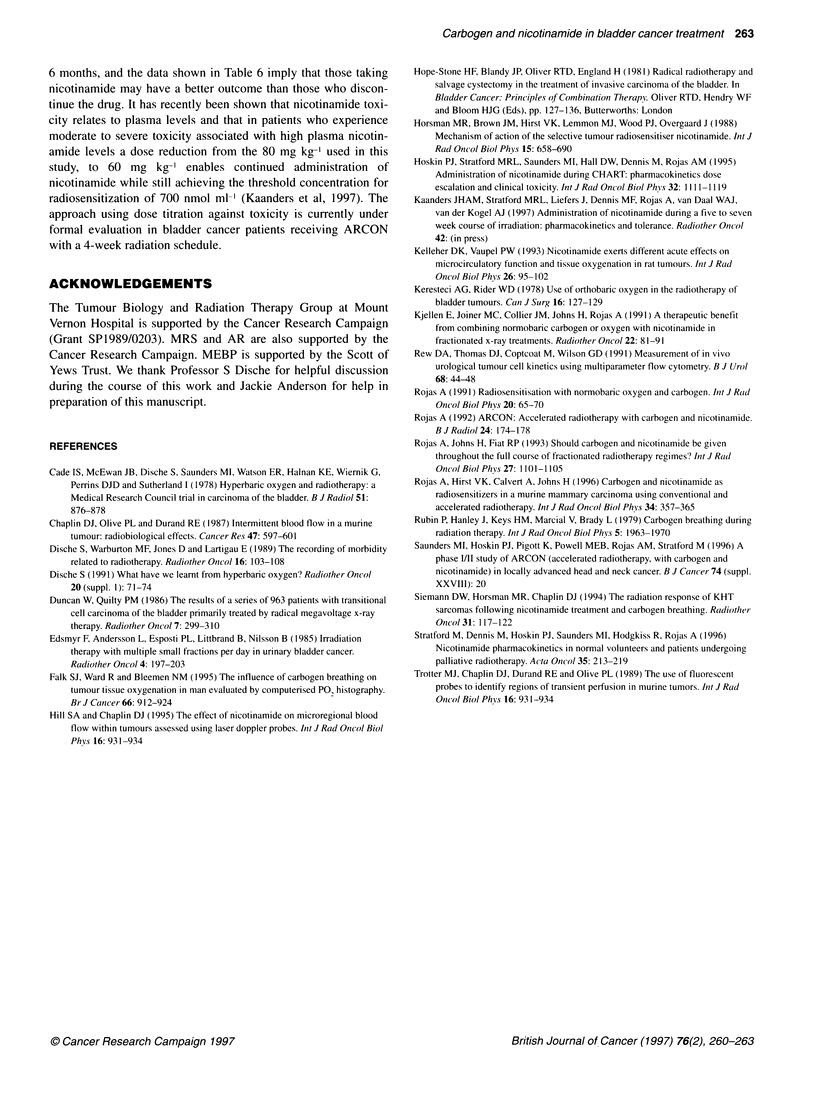# Carbogen and nicotinamide in the treatment of bladder cancer with radical radiotherapy.

**DOI:** 10.1038/bjc.1997.372

**Published:** 1997

**Authors:** P. J. Hoskin, M. I. Saunders, H. Phillips, H. Cladd, M. E. Powell, K. Goodchild, M. R. Stratford, A. Rojas

**Affiliations:** CRC Tumour Biology and Radiation Research Group, Mount Vernon Hospital, Middlesex, UK.

## Abstract

Carbogen and nicotinamide have been evaluated in a phase II study as hypoxia-modifying agents during radical radiotherapy for bladder cancer using a standard daily 20-fraction schedule. Three groups of patients have received (a) nicotinamide alone, given orally in a dose of 80 mg kg(-1) daily with 52.5 Gy in 20 fractions over 4 weeks, (b) carbogen alone, with 50 Gy in 20 fractions over 4 weeks, and (c) carbogen and nicotinamide, with 50-52.5 Gy in 20 fractions over 4 weeks. Ten patients were treated in each group. All patients completed carbogen and radiotherapy as prescribed, but only 45% completed daily nicotinamide over the 4-week treatment period. The end points of this study were acute bowel and bladder morbidity and local control at cystoscopy 6 months after treatment. An expected level of acute bowel and bladder morbidity was seen that reverted to normal in most patients by 12 weeks with no difference between the three treatment groups. Complete response rates at 6 months were seven out of ten (100%) in the nicotinamide alone group, nine out of ten (90%) in the carbogen alone group and seven out of ten (70%) in the carbogen and nicotinamide group. It is concluded that carbogen and nicotinamide may improve the results of daily fractionated radiotherapy in bladder cancer and that further evaluation is required.


British Journal of Cancer (1997) 76(2), 260–263
© 1997 Cancer Research Campaign

### Carbogen and nicotinamide in the treatment of bladder cancer with radical radiotherapy

**PJ Hoskin[1], MI Saunders[1], H Phillips[1], H Cladd[1], MEB Powell[1], K Goodchild[1], MR Stratford[2] and A Rojas[2]**

[1]CRC Tumour Biology and Radiation Research Group, Marie Curie Research Wing, and [2]Gray Laboratory, PO Box 100, Mount Vernon Hospital, Rickmansworth Road, Northwood, Middlesex, HA6 2RJ UK

**Summary** Carbogen and nicotinamide have been evaluated in a phase II study as hypoxia-modifying agents during radical radiotherapy for bladder cancer using a standard daily 20-fraction schedule. Three groups of patients have received (a) nicotinamide alone, given orally in a dose of 80 mg $kg^{-1}$ daily with 52.5 Gy in 20 fractions over 4 weeks, (b) carbogen alone, with 50 Gy in 20 fractions over 4 weeks, and (c) carbogen and nicotinamide, with 50–52.5 Gy in 20 fractions over 4 weeks. Ten patients were treated in each group. All patients completed carbogen and radiotherapy as prescribed, but only 45% completed daily nicotinamide over the 4-week treatment period. The end points of this study were acute bowel and bladder morbidity and local control at cystoscopy 6 months after treatment. An expected level of acute bowel and bladder morbidity was seen that reverted to normal in most patients by 12 weeks with no difference between the three treatment groups. Complete response rates at 6 months were seven out of ten (100%) in the nicotinamide alone group, nine out of ten (90%) in the carbogen alone group and seven out of ten (70%) in the carbogen and nicotinamide group. It is concluded that carbogen and nicotinamide may improve the results of daily fractionated radiotherapy in bladder cancer and that further evaluation is required.

**Keywords:** bladder cancer; hypoxia; nicotinamide; carbogen

Radical radiotherapy is an important treatment for the management of muscle-invasive (T2, T3) and high-grade superficial (T1G3) bladder cancer. It is not, however, universally effective, and 5-year survival ranges from 30% to 50%, with local failure being a major predictor of survival (Hope-Stone et al, 1981; Duncan and Quilty, 1986). A dose–response relationship for bladder carcinoma has been demonstrated using a hyperfractionated schedule to 84 Gy, but even in this series, despite a 62% complete remission rate at 6 months, the 5-year survival was only 37% (Edsmyr et al, 1985).

Possible mechanisms of radioresistance may include hypoxia, repopulation and intrinsic radioresistance. The limited data available have shown that bladder cancer has a median potential cell-doubling time of 17 days (Rew et al, 1991), which suggests that repopulation is probably not a major feature in the failure of radiotherapy to control bladder cancer. Intrinsic radioresistance might be expected to be modified by dose escalation, and this has been demonstrated in the data quoted above. Despite dose escalation, however, a significant failure rate was still seen, and 38% of patients failed ever to achieve local control. This implies that hypoxia may also be important in the control of bladder cancer. Previous studies of hyperbaric oxygen and carbogen have been unsuccessful in improving response rates relative to control arms without hypoxic modification, but these studies can be criticized in terms of the dose fractionation schedules employed (Cade et al, 1978) or the means of carbogen administration (Keresteci and Rider, 1973).

In experimental models, there is evidence to support the use of normobaric oxygen in the form of carbogen (95% oxygen, 5% carbon dioxide) as a hypoxic cell sensitizer (Rojas et al, 1990). The change in tumour $pO_2$ with carbogen is dependent upon the duration of carbogen breathing. It has been shown in one study, using the Eppendorf electrode to measure intratumoral $pO_2$, that this rises to a maximum within the first 10 min of carbogen breathing, falling to baseline within 20 min (Falk et al, 1995). For this reason early clinical trials using 1–2 h of prebreathing with carbogen (Kestereci and Rider 1973; Rubin et al, 1979) cannot be expected to have tested the potential value of carbogen as a hypoxic modifier.

Nicotinamide is the amide of vitamin $B_3$. Acute hypoxia within tumours arises from intermittent closure of blood vessels, resulting in fluctuations in the tumour microcirculation (Chaplin et al, 1987; Trotter et al, 1989). Nicotinamide overcomes acute hypoxia by reducing these changes in the microcirculation (Kelleher and Vaupel, 1993; Hill and Chaplin, 1995). Furthermore, when nicotinamide is combined with carbogen, additional tumour sensitivity to radiation has been demonstrated, with overall enhancement ratios of between 1.8 and 2.1 in animal models using clinically relevant dose schedules of 2 Gy $day^{-1}$ (Kjellen et al, 1991; Rojas et al, 1993). It has therefore been proposed that the combination of carbogen and nicotinamide provides the optimal means of overcoming tumour hypoxia. When combined with acceleration of the radiation schedule, this treatment has been termed ARCON (accelerated radiotherapy carbogen and nicotinamide) (Rojas, 1992). The feasibility of ARCON treatment for carcinoma of the bladder has been evaluated in a formal phase II programme.

*Received 20 November 1996*
*Revised 18 February 1997*
*Accepted 21 February 1997*

*Correspondence to:* PJ Hoskin

### PATIENTS AND METHOD

Between January 1994 and the present time, sequential patients referred for radical radiotherapy for bladder carcinoma have been entered into a phase II study evaluating nicotinamide as a single

**Table 1** Patient details

| | Nicotinamide (52.5 Gy/20 fractions) | Carbogen (50 Gy/20 fractions) | NIC/CARB (50–52.5 Gy/20 fractions) |
|---|---|---|---|
| Number | 10 | 10 | 10 |
| Age (median) | 66 | 69 | 69 |
| Male/female | 9:1 | 10:0 | 8:2 |
| Stage | | | |
| T1 | 1 | 1 | 1 |
| T2 | 3 | 2 | 2 |
| T3 | 6 | 7 | 7 |

**Table 2** Urinary frequency. Number of patients with urinary frequency hourly or more

| Time from day 1 radiotherapy weeks | Nicotinamide | Carbogen | NIC/CARB |
|---|---|---|---|
| 4 | 6 | 5 | 5 |
| 12 | 2 | 2 | 1 |

**Table 3** Bowel function. Bowel motions per day (24 h): median (range)

| Time from day 1 radiotherapy (weeks) | Nicotinamide | Carbogen | NIC/CARB |
|---|---|---|---|
| 4 | 4 (1–6) | 3 (1–6) | 2.5 (2–14) |
| 12 | 1 (1–4) | 2 (1–5) | 1 (1–3) |

**Table 4** Local control at cystoscopy 6 months after radiotherapy

| | Nicotinamide | Carbogen | NIC/CARB |
|---|---|---|---|
| Intercurrent death | | | |
| From bladder cancer | 1 | 0 | 2 |
| Other cause | 0 | 1 | 0 |
| Lost to follow-up | 0 | 0 | 1 |
| Assessable | 9 | 9 | 7 |
| Complete remission | 7 | 9 | 7 |
| Per cent response by intention to treat | 70 | 90 | 70 |

agent, carbogen as a single agent and the combination of nicotinamide and carbogen. This report includes the first 30 of these patients. Patients were selected for radical radiotherapy by virtue of having high-grade superficial bladder carcinoma (T1G3) or muscle-invasive bladder carcinoma (T2, T3a or T3b). All patients had pretreatment staging, computerized tomography (CT) scan and chest radiograph together with routine blood tests, including liver function tests. Only patients with no evidence of disease beyond the bladder or perivesical tissues were entered into the protocol for radical radiotherapy.

Three sequential cohorts of patients received treatment. The first ten patients received nicotinamide alone at a dose of 80 mg $kg^{-1}$. The second group of ten patients received carbogen and the third group of patients both nicotinamide and carbogen. Demographic details including age, sex and tumour stage are shown in Table 1.

Radiation planning and delivery was standard in all patients. The tumour volume was defined using CT planning to cover the bladder with a 2-cm margin. Patients were planned and treated with the bladder empty and a three-field plan was produced with 6- or 15-MV photons. The treatment prescription for the nicotinamide group was 52.5 Gy minimum tumour dose in 20 daily fractions over 4 weeks. The dose was reduced in the carbogen alone group to 50 Gy in 20 daily fractions over 4 weeks because of the concern regarding possible bowel sensitization with carbogen, based on experience with hyperbaric oxygen (Dische 1991). The first five ARCON patients also received the lower dose of 50 Gy, which was increased to 52.5 Gy for the remaining five patients. Plans were produced to cover the target volume with the 90% isodose using normalization to the ICRU maximum isodose (100%).

Nicotinamide was administered at a dose of 80 mg $kg^{-1}$ on the basis of our previous pharmocokinetic work and administered orally 1.5 h before the time of radiation delivery (Hoskin et al, 1995; Stratford et al, 1996). Rapid-release nicotinamide tablets 500 mg and 1 g (Larkhall) were used for all patients. Random peak blood levels were measured by high-performance liquid chromatography (HPLC) to assess compliance. Patients were seen weekly during treatment and thereafter until resolution of acute toxicity, when they were seen monthly until 6 months after treatment at the time of their check cystoscopy. At each visit, details of bowel and urinary function were scored according to the Dische scoring system (Dische et al, 1989).

Carbogen was delivered through a closed breathing system using a face mask (Laerdal Medical Systems) and one-way valve at a flow rate of 15 l $min^{-1}$. Carbogen breathing was started 5 min before radiation delivery during the set-up of the patient and continued throughout the radiation delivery.

### RESULTS

Urine function was recorded in terms of urine frequency. Table 2 demonstrates the number of patients with urinary frequency of more than once per hour and it can be seen that in most patients there is a return to less frequent urine function by 12 weeks after treatment. A similar effect of treatment upon frequency of bowel motion is shown in Table 3. Data for late toxicity are as yet not available, but in those patients followed for more than 1 year no late bowel morbidity has emerged. One patient has required a persisting indwelling catheter because of a fibrosed bladder.

The results for local control as measured by response at cystoscopy 6 months after radiotherapy are shown in Table 4. All patients but one in each of the two carbogen cohorts completed carbogen breathing uneventfully. In contrast, however, only 45% of patients completed nicotinamide as prescribed. Details of

© Cancer Research Campaign 1997 

**Table 5** Nicotinamide compliance. Number continuing to take nicotinamide

| | Day 1 | Day 8 | Day 15 | Day 22 | Day 26 |
|---|---|---|---|---|---|
| Full dose | 20 | 12 | 11 | 10 | 9 |
| Reduced dose | 0 | 3 | 3 | 3 | 3 |
| Total | 20 | 15 | 14 | 13 | 12 |
| Per cent compliance (80 mg kg$^{-1}$) | 100 | 60 | 55 | 50 | 45 |
| Per cent compliance (any dose) | 100 | 75 | 70 | 65 | 60 |

**Table 6** Response related to nicotinamide compliance

| | Nicotinamide dose | | |
|---|---|---|---|
| | Full dose | Reduced dose | Discontinued |
| Number of patients (number having carbogen) | 9 (6) | 3 (1) | 8 (3) |
| Complete response at 6 months (number having carbogen) | 8 (5) | 2 (1) | 4 (1) |
| Per cent response | 89 | 67 | 50 |

nicotinamide compliance are shown in Table 5. The principal reason for nicotinamide intolerance was persistent nausea leading to vomiting despite the administration of regular anti-emetics, including dexamethasone, cyclizine, metoclopromide or ondansetron. The effect of nicotinamide compliance on tumour control is shown in Table 6. Plasma levels of nicotinamide were measured in 14 patients: five due to receive nicotinamide alone and nine in the ARCON group. The results for plasma nicotinamide levels taken at 1 h after oral administration, approximately 30 min before radiotherapy, are shown in Table 7. In two patients non-compliance, otherwise unsuspected, was detected from the plasma levels.

### DISCUSSION

This phase II study has demonstrated that the administration of carbogen with a 4-week radical radiotherapy schedule in the treatment of bladder cancer is feasible. No excess bowel or bladder morbidity has been observed and very high rates of local control are recorded at 6 months. Similar local control rates are seen in all three groups receiving either nicotinamide alone, carbogen alone or the combination of nicotinamide and carbogen.

The administration of nicotinamide on a daily basis over 4 weeks with radical radiotherapy has been troublesome. Around half of patients have not tolerated the medication and have felt unable to continue with it. This occurs at differing times through the 4-week schedule, with no clear pattern emerging, although most patients having early problems discontinue nicotinamide within the first week. The nausea experienced is a particularly intractable symptom that, unlike chemotherapy or radiotherapy-induced nausea and vomiting, does not seem to respond well to conventional antiemetic drugs. These observations are consistent with those noted in our previous volunteer and patient phase I studies (Stratford et al, 1996) and our experience in patients with head and neck cancer receiving ARCON (Saunders et al, 1996). The impact of nicotinamide on tumour control assays in animal models varies according to the tumour type. In the mouse mammary carcinoma CaNT, tumour nicotinamide increases the enhancement ratio with carbogen alone from 1.5 to 1.7 during conventional fractionation (Rojas et al, 1996), but in the KHT sarcoma model an effect as great as carbogen alone is seen with nicotinamide alone, each achieving an enhancement ratio of 1.9 (Siemann et al, 1994), with no increase when the two are combined. Analysis of those patients who completed nicotinamide compared with those who did not, as shown in Table 6, suggests that nicotinamide compliance may predict for a better outcome, although interpretation of such small subgroups can at best only show a trend.

The radiation schedule used in this pilot study is one that implies modest acceleration compared with the conventional 6–6.5 week schedule delivering 60–65 Gy. It is, however, one that has been used at both this institution and other major centres, which report 6-month local control rates of between 45% and 58%. The overall figure of 77% (23 out of 30) noted in this series is therefore encouraging but must be interpreted with caution in view of the small numbers involved. Nonetheless, in the absence of excess morbidity, these data make a strong argument for pursuing carbogen as delivered here in a phase III randomized trial. Despite the poor compliance, patients receiving nicotinamide alone also had a high rate of response, with 70% in complete remission at

**Table 7** Plasma nicotinamide levels in samples taken one hour after oral administration of 80 mg kg$^{-1}$ nicotinamide

| Patient | Nicotinamide-only group (concentration, nmol ml$^{-1}$) | Day of sampling[a] | ARCON group (concentration, nmol ml$^{-1}$) | Day of sampling[a] |
|---|---|---|---|---|
| 1 | 719 | 7 | 0[b] | 7 |
| 2 | 1054 | 15 | 1194 | 7 |
| 3 | 1013 | 7 | 1416 | 8 |
| 4 | 0[b] | 15 | 1247 | 7 |
| 5 | 1368 | 5 | 1615 | 7 |
| 6 | – | | 1578 | 7 |
| 7 | – | | 1698 | 7 |
| 8 | – | | 1722 | 8 |
| 9 | – | | 1813 | 7 |
| 10 | – | | – | |

[a]Time from day 1 of radiotherapy; [b]Reflects non-compliance despite claims of patient; –, plasma sample not collected.

© Cancer Research Campaign 1997

6 months, and the data shown in Table 6 imply that those taking nicotinamide may have a better outcome than those who discontinue the drug. It has recently been shown that nicotinamide toxicity relates to plasma levels and that in patients who experience moderate to severe toxicity associated with high plasma nicotinamide levels a dose reduction from the 80 mg $kg^{-1}$ used in this study, to 60 mg $kg^{-1}$ enables continued administration of nicotinamide while still achieving the threshold concentration for radiosensitization of 700 nmol $ml^{-1}$ (Kaanders et al, 1997). The approach using dose titration against toxicity is currently under formal evaluation in bladder cancer patients receiving ARCON with a 4-week radiation schedule.

### ACKNOWLEDGEMENTS

The Tumour Biology and Radiation Therapy Group at Mount Vernon Hospital is supported by the Cancer Research Campaign (Grant SP1989/0203). MRS and AR are also supported by the Cancer Research Campaign. MEBP is supported by the Scott of Yews Trust. We thank Professor S Dische for helpful discussion during the course of this work and Jackie Anderson for help in preparation of this manuscript.

### REFERENCES

Cade IS, McEwan JB, Dische S, Saunders MI, Watson ER, Halnan KE, Wiernik G, Perrins DJD and Sutherland I (1978) Hyperbaric oxygen and radiotherapy: a Medical Research Council trial in carcinoma of the bladder. *B J Radiol* **51**: 876–878

Chaplin DJ, Olive PL and Durand RE (1987) Intermittent blood flow in a murine tumour: radiobiological effects. *Cancer Res* **47**: 597–601

Dische S, Warburton MF, Jones D and Lartigau E (1989) The recording of morbidity related to radiotherapy. *Radiother Oncol* **16**: 103–108

Dische S (1991) What have we learnt from hyperbaric oxygen? *Radiother Oncol* **20** (suppl. 1): 71–74

Duncan W, Quilty PM (1986) The results of a series of 963 patients with transitional cell carcinoma of the bladder primarily treated by radical megavoltage x-ray therapy. *Radiother Oncol* **7**: 299–310

Edsmyr F, Andersson L, Esposti PL, Littbrand B, Nilsson B (1985) Irradiation therapy with multiple small fractions per day in urinary bladder cancer. *Radiother Oncol* **4**: 197–203

Falk SJ, Ward R and Bleemen NM (1995) The influence of carbogen breathing on tumour tissue oxygenation in man evaluated by computerised $PO_2$ histography. *Br J Cancer* **66**: 912–924

Hill SA and Chaplin DJ (1995) The effect of nicotinamide on microregional blood flow within tumours assessed using laser doppler probes. *Int J Rad Oncol Biol Phys* **16**: 931–934

Hope-Stone HF, Blandy JP, Oliver RTD, England H (1981) Radical radiotherapy and salvage cystectomy in the treatment of invasive carcinoma of the bladder. In *Bladder Cancer: Principles of Combination Therapy*. Oliver RTD, Hendry WF and Bloom HJG (Eds), pp. 127–136, Butterworths: London

Horsman MR, Brown JM, Hirst VK, Lemmon MJ, Wood PJ, Overgaard J (1988) Mechanism of action of the selective tumour radiosensitiser nicotinamide. *Int J Rad Oncol Biol Phys* **15**: 658–690

Hoskin PJ, Stratford MRL, Saunders MI, Hall DW, Dennis M, Rojas AM (1995) Administration of nicotinamide during CHART: pharmacokinetics dose escalation and clinical toxicity. *Int J Rad Oncol Biol Phys* **32**: 1111–1119

Kaanders JHAM, Stratford MRL, Liefers J, Dennis MF, Rojas A, van Daal WAJ, van der Kogel AJ (1997) Administration of nicotinamide during a five to seven week course of irradiation: pharmacokinetics and tolerance. *Radiother Oncol* **42**: (in press)

Kelleher DK, Vaupel PW (1993) Nicotinamide exerts different acute effects on microcirculatory function and tissue oxygenation in rat tumours. *Int J Rad Oncol Biol Phys* **26**: 95–102

Keresteci AG, Rider WD (1978) Use of orthobaric oxygen in the radiotherapy of bladder tumours. *Can J Surg* **16**: 127–129

Kjellen E, Joiner MC, Collier JM, Johns H, Rojas A (1991) A therapeutic benefit from combining normobaric carbogen or oxygen with nicotinamide in fractionated x-ray treatments. *Radiother Oncol* **22**: 81–91

Rew DA, Thomas DJ, Coptcoat M, Wilson GD (1991) Measurement of in vivo urological tumour cell kinetics using multiparameter flow cytometry. *B J Urol* **68**: 44–48

Rojas A (1991) Radiosensitisation with normobaric oxygen and carbogen. *Int J Rad Oncol Biol Phys* **20**: 65–70

Rojas A (1992) ARCON: Accelerated radiotherapy with carbogen and nicotinamide. *B J Radiol* **24**: 174–178

Rojas A, Johns H, Fiat RP (1993) Should carbogen and nicotinamide be given throughout the full course of fractionated radiotherapy regimes? *Int J Rad Oncol Biol Phys* **27**: 1101–1105

Rojas A, Hirst VK, Calvert A, Johns H (1996) Carbogen and nicotinamide as radiosensitizers in a murine mammary carcinoma using conventional and accelerated radiotherapy. *Int J Rad Oncol Biol Phys* **34**: 357–365

Rubin P, Hanley J, Keys HM, Marcial V, Brady L (1979) Carbogen breathing during radiation therapy. *Int J Rad Oncol Biol Phys* **5**: 1963–1970

Saunders MI, Hoskin PJ, Pigott K, Powell MEB, Rojas AM, Stratford M (1996) A phase I/II study of ARCON (accelerated radiotherapy, with carbogen and nicotinamide) in locally advanced head and neck cancer. *B J Cancer* **74** (suppl. XXVIII): 20

Siemann DW, Horsman MR, Chaplin DJ (1994) The radiation response of KHT sarcomas following nicotinamide treatment and carbogen breathing. *Radiother Oncol* **31**: 117–122

Stratford M, Dennis M, Hoskin PJ, Saunders MI, Hodgkiss R, Rojas A (1996) Nicotinamide pharmacokinetics in normal volunteers and patients undergoing palliative radiotherapy. *Acta Oncol* **35**: 213–219

Trotter MJ, Chaplin DJ, Durand RE and Olive PL (1989) The use of fluorescent probes to identify regions of transient perfusion in murine tumors. *Int J Rad Oncol Biol Phys* **16**: 931–934

© Cancer Research Campaign 1997